# Flavonoids as Potential Drugs for *VPS13*-Dependent Rare Neurodegenerative Diseases

**DOI:** 10.3390/genes11070828

**Published:** 2020-07-21

**Authors:** Piotr Soczewka, Krzysztof Flis, Déborah Tribouillard-Tanvier, Jean-Paul di Rago, Cláudia N. Santos, Regina Menezes, Joanna Kaminska, Teresa Zoladek

**Affiliations:** 1 Institute of Biochemistry and Biophysics, Polish Academy of Sciences, Pawinskiego 5A, 02-106 Warsaw, Poland; psoc@ibb.waw.pl (P.S.); kflis@bp.onet.pl (K.F.); kaminska@ibb.waw.pl (J.K.); 2 CNRS, Institut de Biochimie et Génétique Cellulaires, Bordeaux University, CEDEX, 33077 Bordeaux, France; deborah.tribouillard-tanvier@ibgc.cnrs.fr (D.T.-T.); jp.dirago@ibgc.cnrs.fr (J.-P.d.R.); 3 Institut National de la Santé et de la Recherche Médicale INSERM, 33077 Bordeaux, France; 4 Instituto de Biologia Experimental e Tecnológica, Av. República, Qta. do Marquês, 2780-157 Oeiras, Portugal; claudia.nunes.santos@nms.unl.pt (C.N.S.); rmenezes@ibet.pt (R.M.); 5 CEDOC—Chronic Diseases Research Center, Faculdade de Ciências Médicas, Universidade Nova de Lisboa, Rua Câmara Pestana n° 6, 6-A Edifício CEDOC II, 1150-082 Lisboa, Portugal

**Keywords:** yeast model, neurodegenerative diseases, *VPS13* genes, drug repurposing, luteolin, tolcapone, *FET4* gene, iron, sphingolipid biosynthesis, *csg2*Δ

## Abstract

Several rare neurodegenerative diseases, including chorea acanthocytosis, are caused by mutations in the *VPS13A*–*D* genes. Only symptomatic treatments for these diseases are available. *Saccharomyces cerevisiae* contains a unique *VPS13* gene and the yeast *vps13*Δ mutant has been proven as a suitable model for drug tests. A library of drugs and an in-house library of natural compounds and their derivatives were screened for molecules preventing the growth defect of *vps13*Δ cells on medium with sodium dodecyl sulfate (SDS). Seven polyphenols, including the iron-binding flavone luteolin, were identified. The structure–activity relationship and molecular mechanisms underlying the action of luteolin were characterized. The *FET4* gene, which encodes an iron transporter, was found to be a multicopy suppressor of *vps13*Δ, pointing out the importance of iron in response to SDS stress. The growth defect of *vps13*Δ in SDS-supplemented medium was also alleviated by the addition of iron salts. Suppression did not involve cell antioxidant responses, as chemical antioxidants were not active. Our findings support that luteolin and iron may target the same cellular process, possibly the synthesis of sphingolipids. Unveiling the mechanisms of action of chemical and genetic suppressors of *vps13*Δ may help to better understand *VPS13A*–*D*-dependent pathogenesis and to develop novel therapeutic strategies.

## 1. Introduction

Neurodegenerative diseases (NDs) are a group of age-related diseases characterized by progressive neuronal loss, which manifest in a decline of motor or cognitive function. The most common NDs are Alzheimer’s disease (AD), Parkinson’s disease (PD), and Huntington’s disease (HD). In these diseases, one can observe the presence of protein aggregates which contain amyloid β, α-synuclein, and huntingtin, respectively [1], as well as defects in various cellular processes [2,3,4]. These diseases are among the most investigated NDs. However, some NDs are very rare and attract much less attention. Such an ND is chorea-acanthocytosis (ChAc), with about 1000 cases estimated worldwide [5]. ChAc is caused by mutations in *VPS13A* gene [6,7], one of the four *VPS13* homologs present in the human genome [8]; about 90% of these cause a lack of the VPS13A (or chorein) protein [9]. Mutations in other *VPS13* (*B*–*D*) genes have also been linked to different diseases with neurological defects [10,11,12,13,14]. Currently, there is no cure for *VPS13*-associated diseases, only symptomatic treatment is offered. The state of knowledge about these diseases is relatively low and their pathological mechanisms remain unknown [15,16].

To investigate NDs and to find effective therapies, studies on model organisms, such as the yeast *Saccharomyces cerevisiae*, have been carried out [17]. Yeasts enable research approaches unavailable in higher eukaryotes, such as large-scale genetic screening or functional genomics [18]. Yeast models of most NDs express a pathological variant or overexpress a gene associated with a disease [17,19]. Such models can be effectively used to better understand the diseases, identify molecular targets suitable for drug treatment, and select protective molecules [20]. Some of these molecules have been shown to be active in cell culture models and patient-derived cells [21,22,23,24,25]. A study using a PD yeast model identified flavonoids as an active group of compounds against α-synuclein toxicity [23].

Flavonoids are a diverse group of chemical compounds found in plants. The core of flavonoids consists of two benzene rings linked by a heterocyclic pyranic ring. Their diversity results from numerous combinations of types, number, and locations of functional groups bound to the core, which influences their biological activity [26,27]. Flavonoids exhibit antioxidant, antiviral, antibacterial, anti-inflammatory, and anticancer activities [26,27,28,29,30]. Some flavonoids have also shown neuroprotective properties in models of AD and PD, thus being promising drugs for use in clinical trials of NDs [31,32]. The biological effects of flavonoids result from different mechanisms of action, such as the modulation of signaling pathways, antioxidant systems, and metal chelation actions, including iron [27,33].

Iron is necessary for many vital metabolic processes and is the substrate of ROS-generating Fenton reactions. It is an essential cofactor of hemeproteins and Fe-S cluster-containing enzymes, which take part in various cellular processes such as respiration and lipid biosynthesis [34]. Iron homeostasis in yeast is regulated by the transcription factors Aft1 and Yap5, which respond to low and excessive iron availability, respectively [35,36]. Aft1 induces the transcription of genes encoding the components of iron uptake and regulating intracellular iron transport and storage [37]. Depending on iron availability, different iron uptake systems are used by cells. In iron-depleted conditions, yeast cells rely on high-affinity iron uptake systems consisting of Fet3 oxidase and Ftr1 permease. If iron is abundant, uptake is mediated by the low-affinity and low-specificity Fet4 transporter [38,39,40]. Another mechanism of iron uptake is the import of iron-siderophore chelates by siderophore transporters belonging to the Arn/Sit family of proteins [37].

As *S. cerevisiae* contains a homolog of the disease-associated *VPS13A–D* genes, disease models can be created by its deletion or by introducing mutations corresponding to the ones found in patients. These approaches have been used for modelling and studying diseases associated with *VPS13* genes in previous studies [41,42,43]. The yeast mutant devoid of the *VPS13* gene (*vps13*Δ), as well as the *vps13-I2749R* strain—carrying a mutation in *VPS13* corresponding to one found in a ChAc patient—were viable but showed defects in protein trafficking [9]. We have recently found that both *vps13* mutant cells are hypersensitive to very low amounts of the detergent sodium dodecyl sulphate (SDS) [43]. We used this phenotype to screen for multicopy suppressor genes and identified the fragment of the *MYO3* gene encoding type I myosin and other genes. The growth defect of the *vps13*Δ deletion mutant can also be suppressed by FK506, which inhibits calcineurin, a calcium-dependent phosphatase [43]. Therefore, the *vps13*Δ strain can be used as a simple model for *VPS13*-associated diseases, whereas the chemical suppression of *vps13*Δ growth defects can be explored as a tool for drug screening.

In this study, we searched for compounds restoring the *vps13*Δ growth defect in the presence of SDS. The Prestwick library of FDA-approved drugs and an in-house library of natural compounds and their derivatives were screened, leading to the identification of tolcapone and flavonoids, including luteolin, as active chemical suppressors of *vps13*Δ growth defects. Furthermore, these studies allowed for the identification of the structural determinants of their activity. We also found *FET4* to be a multicopy suppressor of *vps13*Δ. Functional and genetic interactions of luteolin, iron compounds, and mutations affecting iron homeostasis and sphingolipid synthesis were also investigated, in order to unveil the possible mechanisms of action of these compounds.

## 2. Materials and Methods

### 2.1. Strains, Media, and Growth Conditions

The *E. coli* strain DH5α was used for plasmid propagation. The *S. cerevisiae* strains used in this study are listed in Table S1. YPD complete medium (1% yeast extract, 2% tryptone, 2% glucose, 1% adenine) or synthetic SC medium (0.067% yeast nitrogen base without amino acids, 2% glucose) with desired supplements (adenine, uracil, amino acids) were used for yeast cultivation. Growth experiments on media with SDS addition were performed using several dilutions of SDS stock solution and images were taken after different time durations, in order to show the most representative and informative results. This experimental approach was necessary, due to the high sensitivity of mutant cells to small changes of final SDS concentration in the plate, which strongly depends on the lot of medium and SDS stock, as well as various physical factors [43]. Plates were incubated at 28 °C. For drop tests, cells were grown overnight in liquid media and cultures were diluted in sterile distilled water to obtain a cell density equal to OD_600_∼1 or OD_600_∼0.1. Subsequently, aliquots of 4-fold serial dilutions of cultures were spotted onto solid media. Chemical compounds added to the YPD medium can be found in Table S2.

KJK181A *vps13*Δ, KJK182 *csg2*Δ *vps13*Δ, and KJK183 *ipt1*Δ *vps13*Δ strains were constructed by deletion of *VPS13* using the *vps13::URA3* cassette. To this end, the strains BY4741, BYcsg2Δ, and BYipt1Δ were transformed with PCR products obtained using pKA475 plasmid (Table S3) as a template and transformants were selected on SC medium without uracil (SC-ura). Genomic integrations were confirmed by PCR on genomic DNA. These strains were used to test SDS sensitivity phenotype and the effect of luteolin and iron salts on cellular growth.

### 2.2. Plasmids

Plasmids used are listed in Table S3. Plasmid pFL44-FET4, bearing a genomic DNA fragment with *FET4* gene, was isolated as a multicopy suppressor of *vps13-I2749R* SDS hypersensitivity phenotype [43] from the pFL-44 genomic bank [44]. The *FET4* gene was amplified by PCR and transferred to YEplac181 [45] and pRS425-P_GPD_ [46] plasmids to obtain YEp181-FET4 and pRS425-P_GPD_-FET4, respectively.

### 2.3. Drug Screening Assay

The assay was based on previously described tests [47,48]. Briefly, *vps13*Δ cells were grown to early exponential phase, OD_600_ was adjusted to 0.5, and 200 µL was spread homogenously onto solid YPD media supplemented with 0.03% SDS (12 × 12 cm square Petri dishes). Sterile filters were placed on the media surface and 2 µL of 10 mM drug solution in dimethyl sulfoxide (DMSO) were applied to each filter disc. In this screen, all 1280 compounds from the Prestwick Chemical Library (Prestwick Chemical, Illkirch-Graffenstaden, France; last used April 2017), the majority of which have been approved by Food and Drug Administration, USA, were tested. DMSO was used as a vehicle control. The plates were incubated at 28 °C for 3 to 5 days. Data were recorded using a Snap Scan1212 (Agfa, Mortsel, Belgium).

### 2.4. Screen of an In-House Library of Natural Compounds and Their Derivatives

The in-house library of natural compounds and their derivatives (Table S2 [49]) was composed from 5 μg/μL stocks in DMSO. An over-night pre-inoculum of *vps13*Δ strain was refreshed and incubated in the shaker at 28 °C for 5 h. Samples corresponding to OD_600_ 0.2 were taken, diluted to a final volume of 250 μL, and spread onto YPD for control and YPD 0.03% SDS medium plates (14 cm round or 12 × 12 cm square). Compounds from the library were applied on filter discs (10, 20, and 30 μg per spot). Mock-treated cells (DMSO solvent only) served as negative control, and luteolin (identified in a drug screen of Prestwick library, described above) as positive control. The plates were incubated at 28 °C for 3 to 5 days. Structure–activity relationship (SAR) analysis was carried out by testing sets of compounds of various structures (Table S2). For SAR, 30 mg of compounds were spotted on filter discs put on YPD, YPD + SDS 0.025%, and YPD + SDS 0.03% solid media with plated *vps13*Δ strain and observed after 3 to 7 days.

## 3. Results

### 3.1. Drug Library Screening Assay Using vps13∆ Strain and SDS Hypersensitivity Phenotype Revealed Luteolin and Tolcapone as Active Compounds

**Figure 1:**
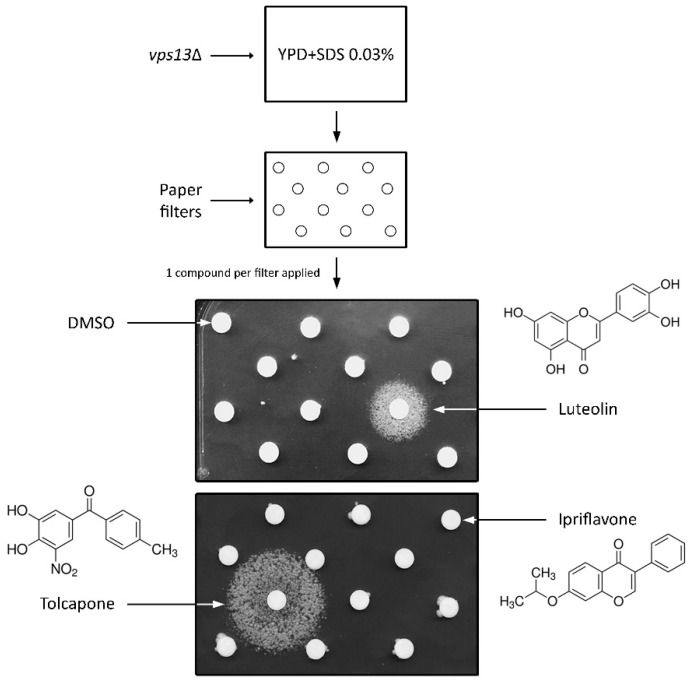
Luteolin and tolcapone are chemical suppressors of *vps13*Δ growth defect. The *vps13*Δ cells were plated on YPD + SDS 0.03%. Compounds from the Prestwick library were applied on filter discs (2 µL of 10 mM solution in DMSO). DMSO was used as a negative control. Plates were incubated for 3 days.

We have previously described that the yeast *vps13*Δ mutant exhibits SDS hypersensitivity and showed that this phenotype has the potential for drug screening [43]. In this study, we adapted this phenotype for drug screening assays (Figure 1) [48], in order to identify molecules preventing the lethality caused by *VPS13* deletion. As SDS is inherently toxic, compounds reducing its toxic properties could also be identified and, thus, the other phenotype—cadmium sensitivity—was subsequently tested (see below). We screened the Prestwick drug repurposing library (Prestwick Chemical) previously used in such screens [48,50], which contains drugs accepted for use in humans. Active compounds were identified after 3–5 days by the observation of growth zones around the filter. Their activity was then confirmed in independent experiments. Seven drugs were identified as suppressors of *vps13*∆ growth defect and two showing some structural similarities were selected for further analysis: luteolin, a naturally occurring flavonoid present in many fruits and vegetables, and tolcapone, a drug used to treat PD, which is a reversible nitrocatechol-type inhibitor of catechol-O-methyltransferase [51] (Figure 1). Another flavonoid present in the Prestwick library, ipriflavone, was not active (Figure 1). To elucidate the structural determinants important for activity, we performed further analyses (see below).

### 3.2. Screen of an In-House Library of Natural Compounds and Their Derivatives Revealed Six Compounds from Two Structural Classes, Flavonoids, and Tannins

**Figure 2:**
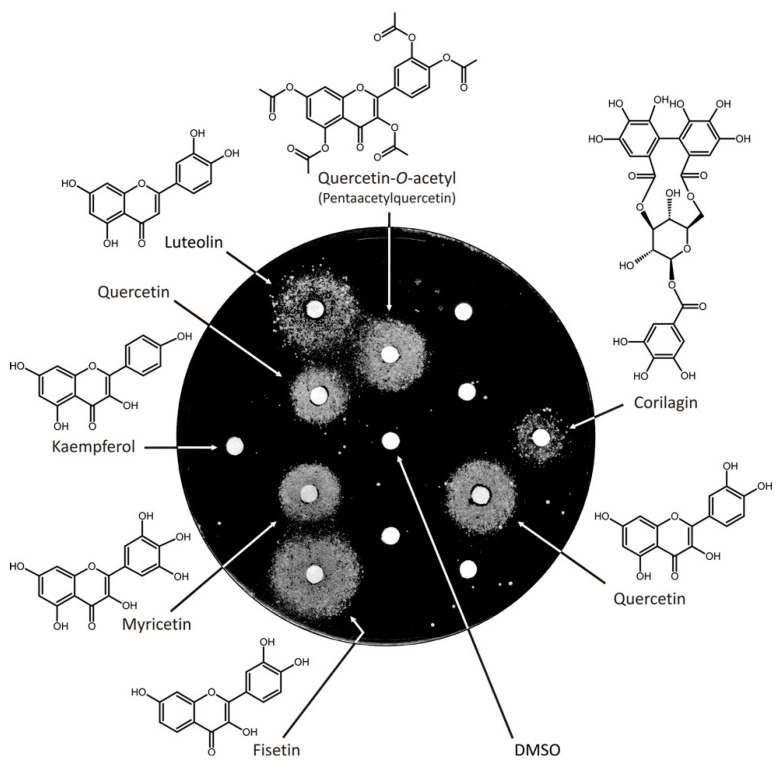
Flavonoids and tannins are chemical suppressors of *vps13*Δ growth defect. The *vps13*∆ strain was plated on YPD + SDS 0.03%. Luteolin was used as a positive control and DMSO as the vehicle control. Kaempferol is indicated as an example of a non-active flavonoid in the conditions used. Compounds were applied at 30 μg per spot. Plates were incubated for 5 days. Two quercetin samples from separate stocks were used.

Identification of luteolin as a suppressor of *vps13*Δ growth defect prompted us to screen an in-house library of natural compounds and their derivatives, in order to evaluate further the efficacy of these compounds as chemical suppressors of *vps13*Δ growth defect in the presence of SDS. Of about fifty compounds tested (Table S2), five—namely, quercetin, pentaacetylquercetin, myricetin, fisetin, and corilagin—were shown to restore *vps13*Δ growth on SDS plates (Figure 2). Among all positive-acting drugs, luteolin caused the largest growth zone (Figure S1). Quercetin, myricetin, and fisetin belong to the group of flavonoids with hydroxyl groups positioned in different carbons of the 3-hydroxyflavone backbone. Pentaacetylquercetin can be deacetylated to quercetin and the activity we observed may have been derived from this active metabolite. Corilagin is a member of the group of hydrolysable tannins, which have a complex structure. Kaempferol, which also belongs to the group of flavonoids, was not active (Figure 2). The identified flavonoids were also active as chemical suppressors of cadmium hypersensitivity of *vps13*Δ cells; furthermore, they also improved the growth of the wild-type strain (Figure S2). Thus, we further focused on SAR analysis of flavonoids.

### 3.3. Analysis of the Structural Determinants of Flavonoids Activity as vps13∆ Suppressors

**Figure 3:**
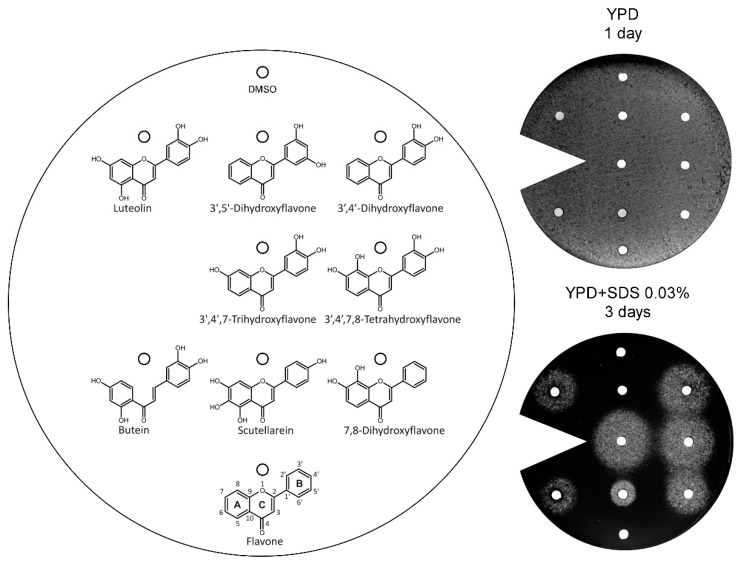
The *vps13*Δ growth-restoring activities of various flavonoids. The *vps13*∆ strain was plated on YPD + SDS 0.03% and YPD for control. The structure of compounds, belonging to different structural subclasses, are shown. Luteolin was used as positive control, and DMSO as negative control. Compounds were applied at 30 μg per spot, according to the diagram. Plates were incubated for 1 or 3 days.

To find determinants of flavonoid structure responsible for the *vps13*∆ chemical suppressor activity, we first tested a set of flavonoids varying in the number and position of hydroxyl groups, such as 3′,5′-dihydroxyflavone, 3′,4′-dihydroxyflavone, 3′,4′,7-trihydroxyflavone, 3′,4′,7,8-tetrahydroxyflavone, butein, scutellarein (4′,5,6,7-tetrahydroxyflavone), and 7,8-dihydroxyflavone. The SAR analysis revealed that two is the minimal number of hydroxyl groups and they must be located on adjacent carbons of the B (preferred) or A ring (Figure 3). In addition to the flavones and flovonols, butein (2′,3,4,4′-tetrahydroxychalcone), an intermediate in the biosynthesis of some flavonoids, was also tested. Although butein does not possess a heterocycle (the corresponding part of the C ring is open), the hydroxyl group positions 3 and 4 are equivalent to positions 3′ and 4′ in flavones. Interestingly, butein was also active (Figure 3), indicating that it is not important whether the C ring is closed or opened, unless other rules are met. These conclusions are also supported by the identification of tolcapone from Prestwick library, which contains A and B rings, but has an open C ring and contains two hydroxyl groups on adjacent carbons (see Figure 1).

**Figure 4:**
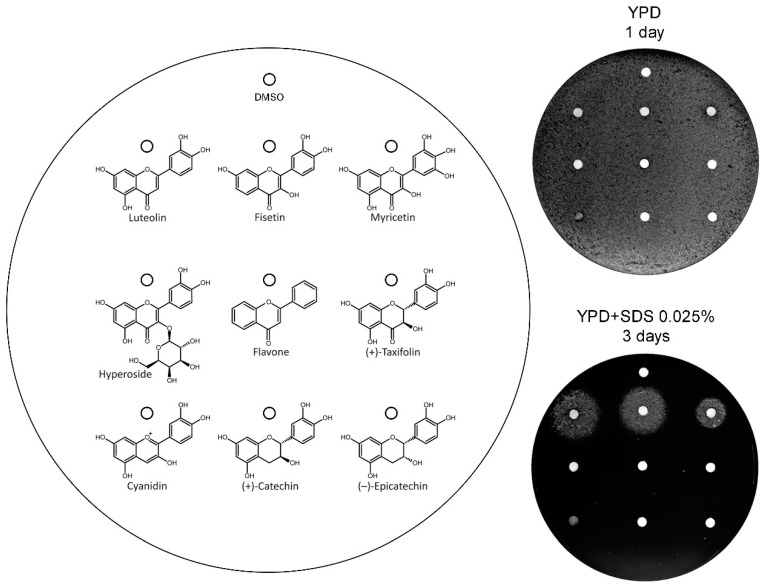
Testing of flavonoids which are hydroxylated in both 3′ and 4′ positions, for their ability to compensate the *vps13*Δ-dependent growth defect. The *vps13*Δ cells were plated on YPD + SDS 0.025% and YPD for control. The structures of compounds are shown. DMSO and flavone were used as negative controls. Compounds were applied at 30 μg per spot, according to the diagram. Plates were incubated for 1 or 3 days.

Then, we asked if other structural elements are important for the activity and tested a second set of flavonoids. This set included luteolin, fisetin, and myricetin, which served as positive controls (see Figure 2); hyperoside (quercetin 3-d-galactoside), which is glycosylated flavonol; taxifolin, cyaniding, catechin and epicatechin, which have various structures with three rings; and flavone as negative control (Figure 4). The analysis showed that the introduction of a glycoside abolished the activity of quercetin. Moreover, the presence of a carbonyl group in the C ring and a double bond between C2 and C3 atoms, in addition to at least two hydroxyl groups in the A or B ring, are also required for activity. These structural features are similar to those previously associated with the antioxidant and metal chelation properties of flavonoids [52,53,54]. However, several antioxidant molecules, including trolox (present in Prestwick library), kaempferol (Figure 2), and catechin (Figure 4), the latter having higher antioxidant potential than luteolin [54], were not active. Thus, the iron chelating property, not antioxidant activity, is possibly important for *vps13*∆ suppression by flavonoids.

### 3.4. The FET4 Gene Encoding Iron Transporter Fet4 Suppresses vps13Δ and Mutations Affecting Iron Transport Result in SDS Hypersensitivity

**Figure 5:**
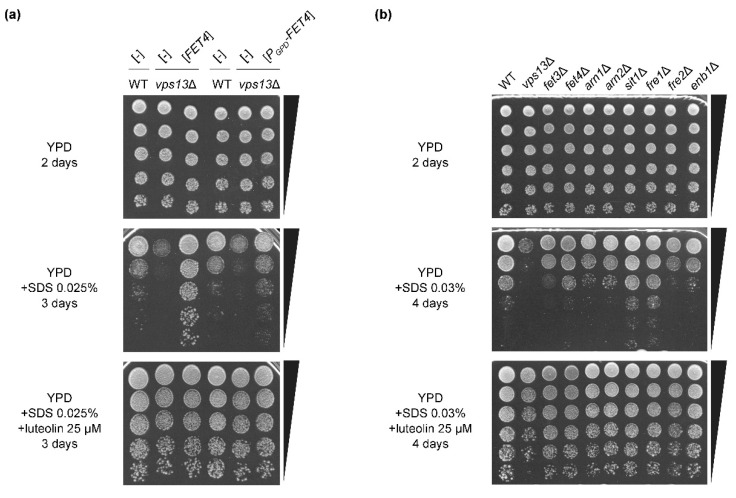
Genetic link between iron transport, luteolin, and SDS hypersensitivity. (**a**) The *vps13*Δ growth defect on SDS media is suppressed by *FET4* overexpression and by luteolin. Growth of *vps13*Δ strain bearing a plasmid expressing *FET4* from native or GPD promoter, or empty vector, was compared with respective controls by drop test on YPD, YPD + SDS 0.025%, and YPD + SDS 0.025% + luteolin 25 µM plates. Images were taken after 2 or 3 days of incubation. (**b**) Mutants defective in iron transport are hypersensitive to SDS; this phenotype is suppressed by luteolin. Growth of *fet3*Δ, *fet4*Δ, *arn1*Δ, *arn2*Δ, *sit1*Δ, *fre1*Δ, *fre2*Δ, and *enb1*Δ strains on YPD, YPD + SDS 0.03%, and YPD + SDS 0.03%+luteolin 25 µM plates was compared with wild-type and *vps13*Δ controls by drop test. Images were taken after 2 or 4 days of incubation.

In a multicopy genomic library screen for suppressor genes of *vps13* SDS hypersensitivity phenotype [43], we identified a plasmid containing the *FET4* gene encoding a low affinity plasma membrane iron (Fe^2+^) transporter [38] (Figure 5a). This finding indicated the importance of iron transport in the response to SDS stress. However, placing the *FET4* gene under the control of a strong constitutive promoter, GPD, caused less efficient suppression, suggesting that *FET4* expression must be tightly controlled by the native promoter (Figure 5a). Addition of luteolin to SDS plates improved *vps13*Δ growth, irrespective of the presence of *FET4*. There seemed to be no cumulative effect of exposing cells to luteolin and overexpression of *FET4*, consistent with the view that both treatments may act on the same pathway. The parental wild-type strain also displayed growth enhancement in response to luteolin, which mitigates SDS toxicity even in the presence of Vps13, but the effect on *vps13*Δ was more pronounced in test conditions. These findings are in line with the view that a moderate increase of iron in *vps13*Δ cells may help to cope with SDS stress.

Further corroboration of the functional association between iron transport and *vps13*Δ growth restoration on SDS-containing plates was provided by the analysis of a set of deletion mutants, devoid of genes encoding iron transporters *fet3*Δ and *fet4*Δ; siderophore transporters *arn1*Δ, *arn2*Δ, *sit1*Δ, and *enb1*Δ; or transport factors *fre1*Δ and *fre2*Δ. Interestingly, *fet3*Δ, *fet4*Δ, *arn1*Δ, *arn2*Δ, *fre2*Δ, and *enb1*Δ mutants appeared to be more sensitive to SDS, as compared to the wild-type strain (Figure 5b), supporting the notion that SDS stress response requires controlled upregulation of iron uptake which apparently does not involve Sit1 siderophore transporter or ferric reductase Fre1, which reduces siderophore-bound iron. Interestingly, the growth of wild-type and these mutant strains was improved by addition of luteolin (Figure 5b). Iron is easily bound by flavonoids [53,55]; thus, luteolin may (directly or indirectly) act by supporting iron transport into the cell, or may affect the same processes for which additional iron is required. Possibly, the impairment of iron transport in response to SDS stress may be the cause of hypersensitivity of *vps13*Δ, which is corrected either by *FET4* upregulation or by luteolin.

### 3.5. Iron Salts and Enterobactin Are Chemical Suppressors of vps13Δ

**Figure 6:**
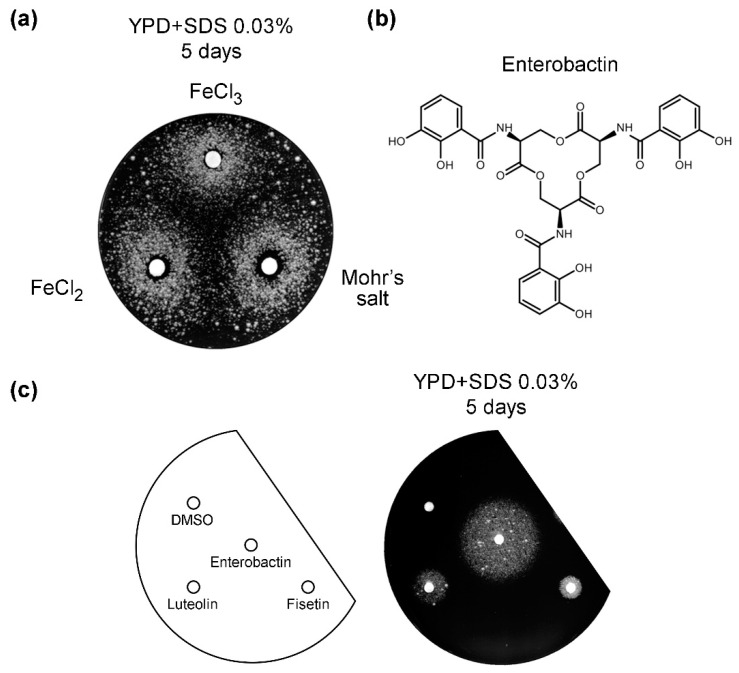
Iron salts and enterobactin restore growth of *vps13*Δ cells on SDS plates. (**a**) The *vps13*∆ strain was plated on YPD + SDS 0.03%. Iron salts (FeCl_3_, FeCl_2_, and Mohr’s salt Fe(NH_4_)_2_(SO_4_)_2_) were applied at 10 µL of 1 M solution per spot. Plates were incubated for 5 days. (**b**) Structure of enterobactin. (**c**) The *vps13*∆ strain was plated on YPD + SDS 0.03%. Enterobactin and flavonoids were applied at 10 μg per spot. Luteolin and fisetin were used as positive controls, and DMSO as negative control. Plates were incubated for 5 days.

To explore the genetic link between iron transport, luteolin, and SDS sensitivity, we tested various iron compounds for their effect on *vps13*Δ growth on SDS-containing plates. Addition of soluble iron salts, namely iron(II)chloride, iron(III)chloride, and iron ammonium sulfate (Mohr’s salt) restored growth of *vps13*∆ cells on SDS plates (Figure 6a). A small zone of inhibition close to the filter was observed, indicating that high iron ion concentrations were toxic. These results may indicate that *vps13*Δ cells require an excess of iron to respond to SDS stress or that they have an iron deficit. An argument against the last possibility is the fact that the transcriptional activation of *FET3* and *CTH2*, which are involved in a signaling pathway responding to iron deficiency [56], was unchanged in *vps13*Δ, compared to the wild-type and upon SDS stress, as tested using the respective *lacZ* reporter fusions (Figure S3a,b). Moreover, the sensitivity of *vps13*Δ to ferrozine, a strong iron chelator, was similar to that of the wild-type strain (Figure S3c), suggesting that iron levels were similar in both strains.

We also tested whether the growth phenotype of *vps13*Δ was affected by enterobactin (also known as enterochelin; Figure 6b), which is a high affinity siderophore belonging to the catechol group of compounds. It is found in bacteria such as *Escherichia coli* and *Salmonella typhimurium*. Enterobactin bound to Fe^3+^ is transported by the Enb1 transporter into yeast cells [57]. Interestingly, enterobactin restored growth of *vps13*Δ on SDS-containing plates (Figure 6c). Enterobactin also contains structural elements, such as two hydroxyl groups on adjacent carbons of the benzene ring and a carbonyl group in the central ring (Figure 6b), which we found important for *vps13*Δ SDS phenotype suppression by flavonoids in the SAR analysis. This suggests that enterobactin and flavonoids, such as luteolin and fisetin, may suppress the growth phenotype of *vps13*Δ by a similar iron-dependent mechanism.

### 3.6. Characterization of the Possible Mechanism of Flavonoids Action

**Figure 7:**
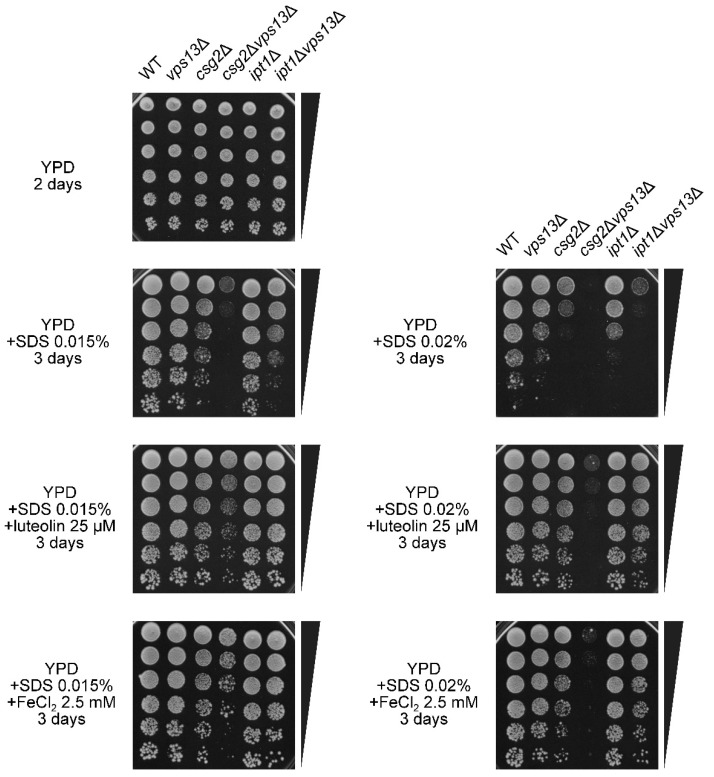
The *ipt1*Δ and *csg2*Δ mutations causing defects in complex sphingolipid biosynthesis are suppressed by luteolin and iron salt, and negatively interact with *vps13*Δ. Growth of wild-type, *vps13*Δ (KJK181A), *csg2*Δ, *ipt1*Δ, and the respective double mutants on YPD plates containing SDS was compared by drop test. The effect of luteolin and FeCl_2_ on growth is also shown. Images were taken after 2 or 3 days of incubation.

The importance of iron supply in the response of *vps13*Δ cells to SDS turned our attention to iron-dependent cellular processes, which might be essential under this stress condition. Iron ions are cofactors of heme proteins, Fe-S clusters proteins, and other enzymes, which take part in several processes, including iron transport and lipid biosynthesis [34]. Lipid biosynthetic pathways, producing unsaturated fatty acids, sterols, and sphingolipids, contain heme proteins. Thus, iron co-ordinates the synthesis of various lipids in response to cell needs. SDS, as a detergent, disrupts the cell wall and plasma membrane; thus, the remodeling of the cell wall and lipid composition of the plasma membrane are crucial in the SDS response [43,58,59]. Sphingolipid biosynthesis has previously been found to be important for SDS tolerance. Cells devoid of *IPT1,* encoding inositolphosphotransferase responsible for the last step in the biosynthesis of the major plasma membrane sphingolipid mannose-(inositol-P)_2_-ceramide [M(IP)2C] [60], and *CSH1* and *SUR1,* encoding mannosylinositol phosphorylceramide (MIPC) synthase catalytic sub-units [61,62], have been shown to be hypersensitive to SDS [58]. Thus, the sensitivity of *csg2*Δ and *ipt1*Δ mutants, defective in complex sphingolipid biosynthesis, to various SDS concentrations, and their genetic interaction with *vps13*Δ mutation in these conditions were tested. The *csg2*Δ strain, devoid of a protein required for the mannosylation of inositolphosphorylceramide (IPC), was more sensitive than *ipt1*Δ to SDS, as *csg2*Δ grew slower while *ipt1*Δ grew at a similar rate to the wild-type strain on YPD + SDS 0.015% (Figure 7). The effect of both these mutations was additive with *vps13*Δ, as *csg2*Δ *vps13*Δ did not grow on YPD + SDS 0.015% and *ipt1*Δ *vps13*Δ stopped growing when the SDS concentration was higher (YPD + SDS 0.02%; Figure 7) while respective single mutants grow in these conditions. These negative genetic interactions suggest that Vps13 contributes to cell survival during SDS stress in parallel to complex sphingolipid biosynthesis pathways. Interestingly, when suppression by luteolin or FeCl_2_ of the respective mutants was studied, it was observed that *csg2*Δ, *ipt1*Δ, and the respective double mutants *csg2*Δ *vps13*Δ and *ipt1*Δ *vps13*Δ were suppressed (Figure 7). Thus, MIPC and M(IP)_2_C, the last two products of the sphingolipid biosynthesis pathway, seemed not to be important in the action of luteolin and FeCl_2_ on *vps13*Δ cells. As *vps13*Δ and *csg2*Δ or *ipt1*Δ showed genetic negative interaction and luteolin or iron suppressed growth defect of single and double mutants, we conclude that there must be a pathway, independent of the Vps13 protein and the synthesis of complex sphingolipids or the upregulation or downregulation of which, by the administration of luteolin or iron, is helpful under SDS stress.

## 4. Discussion

The pathogenesis of neurodegenerative diseases caused by mutations in *VPS13A*-*D* genes is not well understood and no effective treatment is available. Simple model organisms, such as the yeast *S. cerevisiae*, offers opportunities to improve our understanding of cellular pathogenic processes and perform drug repurposing screening. This is possible by using mutations in the yeast *VPS13* gene encoding the Vps13 protein, the orthologue of human *VPS13A-D* gene products [9]. In this paper, we screened the Prestwick library and an in-house library of natural compounds and their derivatives and identified several polyphenols, including the flavone luteolin, for protective compounds overcoming some of the defects caused by Vps13 deficiency. These defects can also be reversed by overexpression of *FET4,* encoding an iron transporter, and the addition of iron salts or iron-binding siderophores to the medium. This indicates the importance of cellular iron concentration for *vps13*Δ cells to survive under stress and provides a link to the metabolic pathway, which we conclude to be involved in the mechanism of luteolin action—the sphingolipid biosynthesis pathway.

Screening of the Prestwick drug repurposing library and an in-house library of natural compounds and their derivatives revealed several polyphenols from the group of flavonoids and tannins as able to overcome the defects caused by Vps13 deficiency. Besides the SDS hypersensitivity phenotype used in the screen, the cadmium hypersensitivity of *vps13* cells was also effectively suppressed, showing that the mechanism of action of the selected compounds was not SDS-specific but more general. However, some transport defects, such as the defect of Sna3-GFP transport to the vacuole [42] were not suppressed (unpublished results). Flavonoids such as luteolin, quercetin, fisetin, and myricetin are best-known for their antioxidant and metal chelation activities [53,54]. Quercetin, together with (−) epigallocatechin-3-gallate, was found previously as a compound which has strong activity to restrain α-synuclein toxicity in a yeast model system of PD [23]. From the SAR analysis, the authors concluded that these compounds act by virtue of antioxidant and metal chelating properties; these conclusions were also supported by the observation that iron salt negatively affected this model system. Our SAR analysis similarly showed that the suppressor activity of flavonoid compounds correlated with the same structural requirements as for their iron-binding and antioxidant properties. However, anthocyanins and anthocyanidins, both very potent antioxidants from another structural group, which were present among the compounds in the in-house library of natural compounds, as well as several other antioxidants that we tested, such as trolox, kaempferol, and catechin [54], were unable to restore the growth of *vps13*Δ cells in the presence of SDS. Altogether, the data suggest that the protective action of flavonoids is rather not related to antioxidant action but, instead, depends on mechanisms linked to their metal-binding properties.

In contrast to the results obtained by testing flavonoids using the yeast model of PD [23], the addition of iron salts showed positive effects in our model system, suggesting that increased cellular levels of iron can help to overcome disturbances caused by Vps13 deficiency. In support of this idea, the iron siderophore enterobactin, which transports iron into cells, was also a very potent suppressor of *vps13*Δ. Moreover, in our genetic screen, the *FET4* gene, encoding a low-affinity iron transporter, was found as a multicopy suppressor of the *vps13*Δ growth defect, showing again that increased iron transport into the cell is helpful in this condition. Interestingly, luteolin restored the growth of *vps13* cells regardless of the presence of multiple copies of *FET4*; thus, the effect of these two factors was not additive, suggesting that they act on the same process. Possible mechanisms of flavonoids and iron action involve binding to specific proteins, affecting metabolic or signaling pathways, or changing gene expression to induce a protective response in cells against SDS stress [63,64].

Increased sensitivity to SDS suggests the defective structure of cellular membranes in the *vps13* mutant. Membranes are composed of a complex mixture of lipids, including sphingolipids. The composition of membrane lipids changes along with the secretory pathway in *S. cerevisiae*: Sphingolipids are generated in the ER and Golgi, then delivered to the plasma membrane where they are most abundant [65] and play a significant role in responding to various stresses, both as building components and as signaling molecules. By using the yeast genetic approach, we have demonstrated the link of Vps13 and luteolin with a sphingolipid pathway. The *ipt1*Δ and *csg2*Δ mutations causing defects in complex sphingolipid biosynthesis are suppressed by luteolin and iron salt, and negatively interact with *vps13*Δ. One may find indirect connections between Vps13 and sphingolipids through the analysis of previously published data. Noteworthy, *vps13*/*soi1* mutants were originally selected in the screen for suppressors of *kex2* mutation disturbing Kex2 protease activity in late Golgi [66] and it was further documented that *vps13* mutation affects retrograde traffic of proteins (such as Vps10 receptor) between endosome and late Golgi [67]. As defects in retrograde vesicular transport between endosomes and Golgi apparatus result in disturbances in sphingolipid biosynthesis [68], this led to the assumption that the *vps13* mutant may have disturbed sphingolipid biosynthesis as well. Moreover, the first step of complex sphingolipid synthesis—the conversion of ceramide to IPC—is essential for the viability of the cell and its regulation involves processing by Kex2 protease [69]. As it has been documented that *vps13* affects wild-type Kex2 cycling between trans Golgi and endosomes [67], *vps13*Δ mutation may negatively affect IPC levels. In addition, examination of reporters for different lipids showed that prospore membranes in *vps13* cells have reduced levels of phosphatidylinositol-4-phosphate (PI(4)P), phosphatidylinositol-4,5-bisphosphate (PI(4,5)P2), and phosphatidic acid [70]. Reduction of the latter two lipids could account for an effect on PI(4)P levels [71,72] which, in turn, may also negatively affect IPC levels [73] in *vps13*Δ cells as well. Sphingolipid homeostasis is regulated by complicated sensing and feedback mechanisms, which involve signaling pathways regulating the L-serine:palmitoyl-CoA acyltransferase (SPT), the first enzyme of the sphingolipid biosynthesis pathway [74,75], and changed response of cells to myriocin—the inhibitor of this enzyme—can indicate disturbances in sphingolipid biosynthesis [68,71,76]. Possible defects in sphingolipids in *vps13* cells which are also resistant to myriocin [68] require further study.

Sphingolipid synthesis is also linked to iron, as high iron levels cause an increase of total sphingolipid precursors, such as long-chain bases (dihydrosphingosine and phytosphingosine) and their phosphate derivatives, where decreasing these levels by myriocin treatment increases yeast tolerance to high iron. Sphingolipids play a signaling role in iron response, as the loss of Pkh1 and Ypk1 kinases [64], which respond to and regulate sphingolipid levels, increases iron tolerance in yeast [77]. We did not observe the hallmark of iron deficiency in *vps13*Δ mutant cells and no iron deficiency was found in the *vps13-D716H* mutant by others [78]; however, increased iron levels in response to SDS might be helpful in stress defense. Interestingly, sphingolipid homeostasis is also a target of luteolin in human cancer cells [79]. Thus, the addition of luteolin or iron salts to the SDS-containing medium may correct defects of Vps13 deficiency and promote growth by affecting the synthesis of sphingolipid precursors.

Dysregulation of sphingolipid metabolism is involved in a great deal of neurodegenerative disorders, manifesting in neurological symptoms as sphingolipids are highly enriched in the nervous system, where they play important regulatory roles. For example, mutations in genes encoding sub-units of SPT have been shown to cause sensory and autonomic neuropathy type 1 (HSAN1) [80]. Defects in retrograde vesicular transport have been shown to cause disturbances in sphingolipid biosynthesis and result in the human neurodegenerative disease cerebello-cerebral atrophy type 2 (PCCA2) [68,81]. Sphingolipid metabolism is also perturbed in AD, PD, and HD. With the emerging role of ceramide and sphingosine-1-phosphate (S1P) as cell-signaling hubs in the pathophysiology of several neurodegenerative diseases, unified therapeutic approaches are possible. It is not known if, in yeast or human cells devoid of VPS13 proteins, the sphingolipid homeostasis is disturbed, which remains to be analyzed. However, deficiency of Vps13A negatively affects the PI(4)P pool, which affects the synthesis of complex sphingolipids in mammalian cells [82]. Thus, it is possible that *VPS13* negatively affects sphingolipid homeostasis and the drugs selected here, tolcapone and flavonoids, alleviated this defect.

## 5. Conclusions

The screening of drug candidates using yeast models of neurodegenerative diseases has already proved efficient to isolate compounds that were later shown to be active in patient-derived cells. This study revealed that tolcapone and several natural flavonoids, including luteolin, exert positive effects on a *vps13*Δ yeast model of *VPS13*-dependent rare neurodegenerative diseases. These compounds may represent candidates for drug repurposing and dietary supplementation as a potential strategy to improve the existing symptomatic treatments of these diseases. Characterization of the mechanism of luteolin action revealed iron homeostasis and sphingolipid biosynthesis as possible targets. Our study also indicates that further research on sphingolipids in cells devoid of the Vps13 protein is critical to better understand the pathogenesis of *VPS13*-dependent rare neurodegenerative diseases and to develop effective therapeutic strategies.

## Supplementary Materials

The following are available online at https://www.mdpi.com/2073-4425/11/7/828/s1, Figure S1: Comparison of chemical suppressors of *vps13*Δ growth defect upon SDS stress; Figure S2: Comparison of the action of flavonoids on wild-type and *vps13* strains upon cadmium stress; Figure S3: Signaling pathway involving *FET3* and *CTH2* genes responding to iron deficiency is not activated in *vps13*Δ cells; Table S1: List of *S. cerevisiae* strains used; Table S2: List of chemical compounds used; Table S3: List of plasmids used.
