# Supplementary material for: Flavonoids as Potential Drugs for VPS13-Dependent Rare Neurodegenerative Diseases
Source: Genes (Basel). 2020 Jul 21;11(7):828. doi: 10.3390/genes11070828 (PMC7397310; doi:10.3390/genes11070828)
Supplement: Supplementary file 1 [file genes-11-00828-s001.pdf]

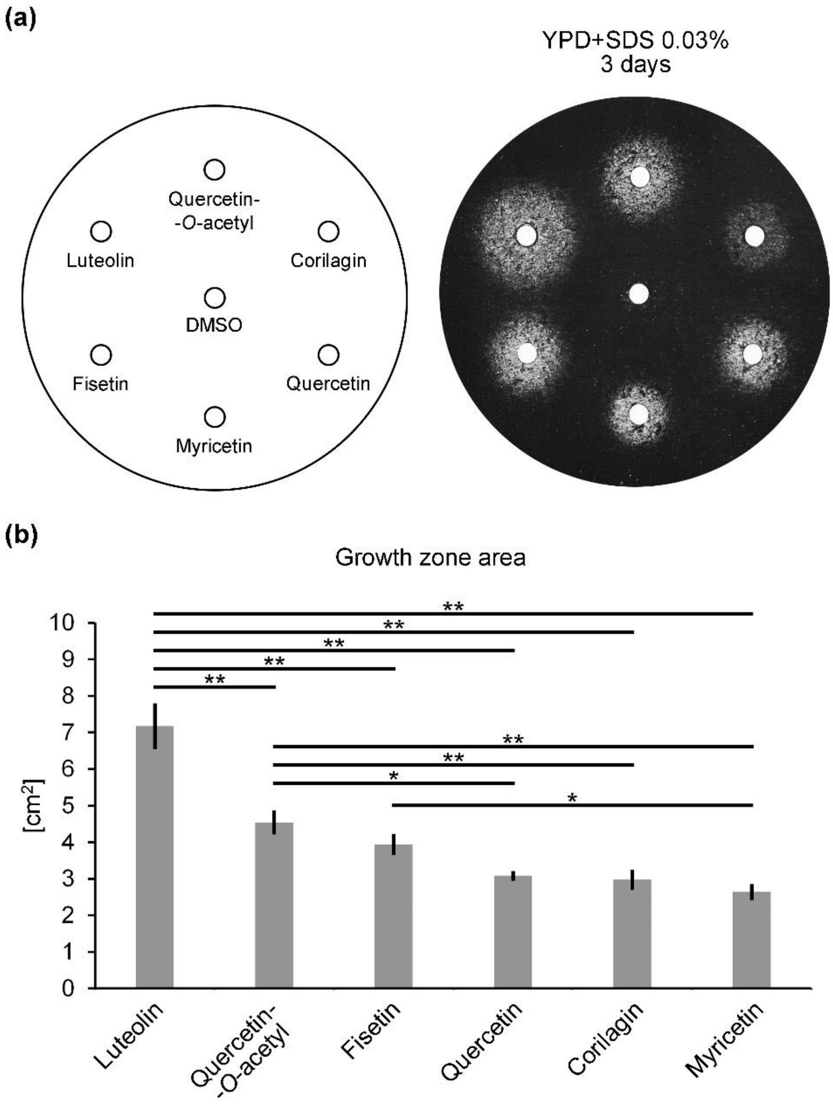

Figure S1. Comparison of chemical suppressors of *vps13*Δ growth defect upon SDS stress. The *vps13*Δ strain was plated on YPD + SDS 0.03%. Compounds were applied on the filter discs (5 µl of 10 mM solutions in DMSO). DMSO was used as a negative control. Plates were incubated for 3 days. The experiment was performed in triplicate and representative results are shown in **(a)** and quantification of growth zone areas in **(b)**. Results were analysed by one-way ANOVA followed by Tukey's multiple-comparisons test (* $p < 0.05$, ** $p < 0.01$). Error bars indicate standard deviation.

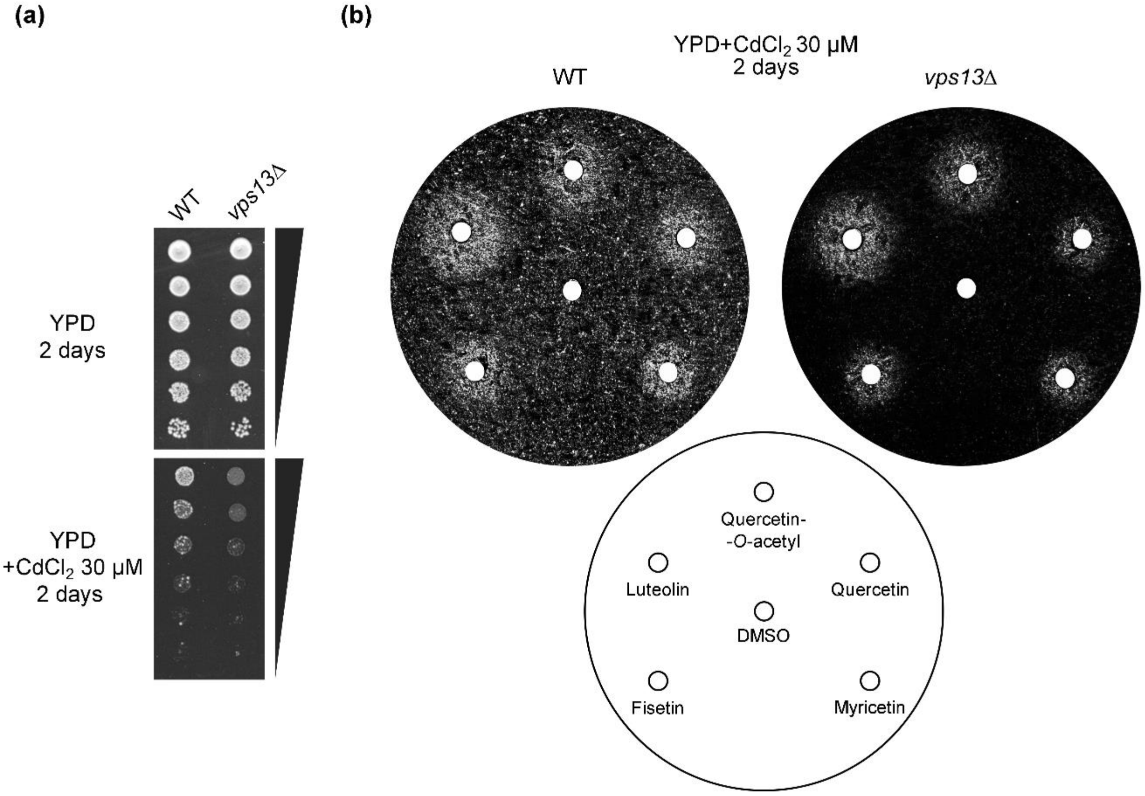

Figure S2. Comparison of actin of flavonoids on wild-type and *vps13*Δ strains upon cadmium stress. **(a)** The growth of wild-type and *vps13*Δ cells in the presence of $CdCl_2$ (30 µM) was compared by drop test. Plates were incubated for 2 days. **(b)** The wild-type and *vps13*Δ cells were plated on YPD + $CdCl_2$. Active flavonoids were applied at amounts of 5 µl of 10 mM solution in DMSO per spot. Plates were incubated for 2 days.

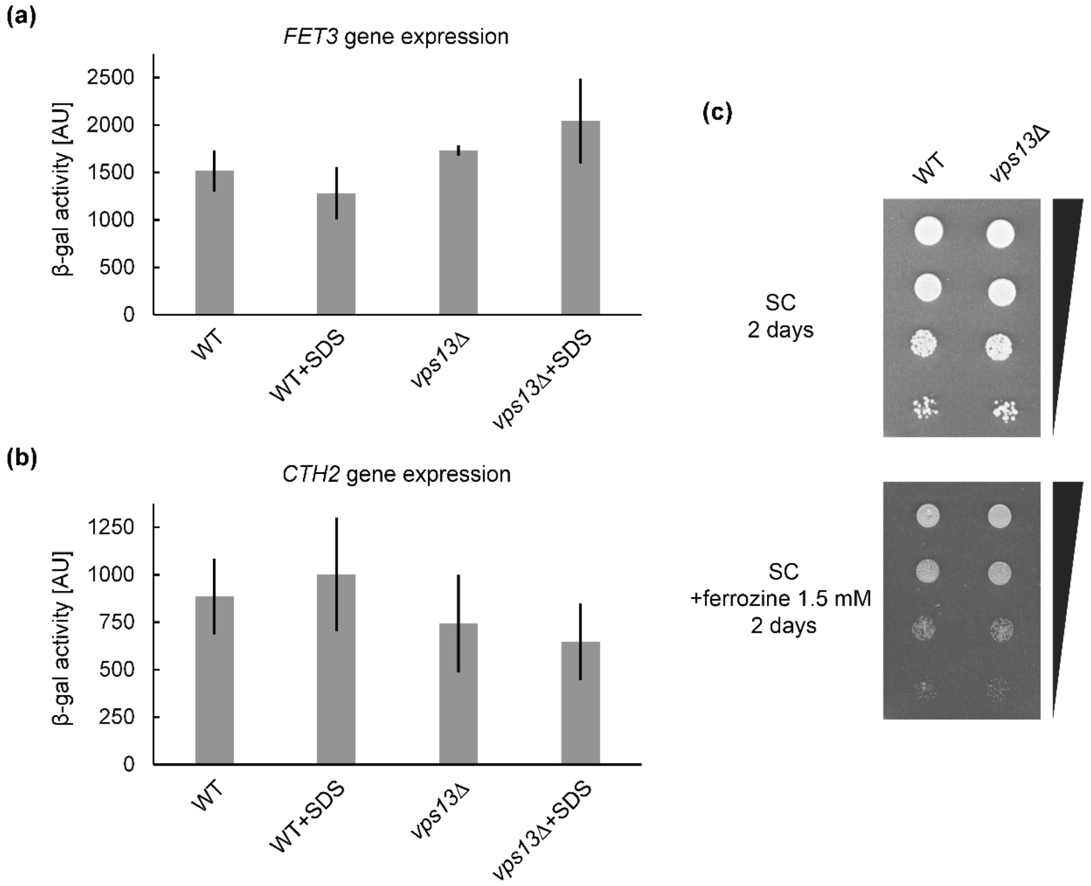

Figure S3. Signaling pathway involving *FET3* and *CTH2* genes responding to iron deficiency is not activated in *vps13*Δ cells. β-galactosidase activity was measured in wild-type and *vps13*Δ cells bearing plasmids with the reporter fusions, *FET3-lacZ* **(a)** or *CTH2-lacZ* **(b)**. For β-galactosidase activity measurements, cells were cultured in SC-ura medium overnight to OD of ~1.5 at 28°C. SDS to a final concentration of 0.005% was added to half of the cultures and incubation proceeded for a further 4 h. Protein extracts were prepared with glass beads. Activity of β-galactosidase was measured in activity units nmol/min/mg of protein [AU], as described previously [43]. Three independent experiments were performed. Results were analyzed by one-way ANOVA (n = 3); p = 0.13 **(a)**, p = 0.52 **(b)**. Error bars indicate standard deviation. **(c)** Growth of wild type and *vps13*Δ cells on SC and SC + ferrozine 1.5 mM was compared by drop test. Images were taken after 2 days of incubation.

Table S1. List of *S. cerevisiae* strains used.

Strain	Genotype	Source or reference
BY4742	*MATα his3Δ1 leu2Δ0 lys2Δ0 ura3Δ0*	Open Biosystem
BY4741	*MAT**a** his3Δ1 leu2Δ0 met15Δ0 ura3Δ0*	Open Biosystem
BYvps13Δ	*MAT**a** his3Δ1 leu2Δ0 met15Δ0 ura3Δ0 vps13::kanMX*	Open Biosystem
KJK181A	*MAT**a** his3Δ1 leu2Δ0 met15Δ0 ura3Δ0 vps13::URA3*	This study
BYfet3Δ	*MAT**a** his3Δ1 leu2Δ0 met15Δ0 ura3Δ0 fet3::kanMX*	Open Biosystem
BYfet4Δ	*MAT**a** his3Δ1 leu2Δ0 met15Δ0 ura3Δ0 fet4::kanMX*	Open Biosystem
BYarn1Δ	*MAT**a** his3Δ1 leu2Δ0 met15Δ0 ura3Δ0 arn1::kanMX*	Open Biosystem
BYarn2Δ	*MAT**a** his3Δ1 leu2Δ0 met15Δ0 ura3Δ0 arn2::kanMX*	Open Biosystem
BYsit1Δ	*MAT**a** his3Δ1 leu2Δ0 met15Δ0 ura3Δ0 sit1::kanMX*	Open Biosystem
BYfre1Δ	*MAT**a** his3Δ1 leu2Δ0 met15Δ0 ura3Δ0 fre1::kanMX*	Open Biosystem
BYfre2Δ	*MAT**a** his3Δ1 leu2Δ0 met15Δ0 ura3Δ0 fre2::kanMX*	Open Biosystem
BYenb1Δ	*MAT**a** his3Δ1 leu2Δ0 met15Δ0 ura3Δ0 enb1::kanMX*	Open Biosystem
BYcsg2Δ	*MAT**a** his3Δ1 leu2Δ0 met15Δ0 ura3Δ0 csg2::kanMX*	Open Biosystem
KJK182	*MAT**a** his3Δ1 leu2Δ0 met15Δ0 ura3Δ0 csg2::kanMX vps13::URA3*	This study
BYipt1Δ	*MAT**a** his3Δ1 leu2Δ0 met15Δ0 ura3Δ0 ipt1::kanMX*	Open Biosystem
KJK183	*MAT**a** his3Δ1 leu2Δ0 met15Δ0 ura3Δ0 ipt1::kanMX vps13::URA3*	This study

Table S2. List of chemical compounds used.

Chemical compound	Source[1]
**In-house library of natural compounds and their derivatives**	
Benzoic acid-4-*O*-β-D-glucuronide	synthesized
Caffeic acid (3,4-Dihydroxycinnamic acid)	commercial
Caffeic acid-3′-*O*-sulfate and Caffeic acid-4′-*O*-sulfate	synthesized
(+)-Catechin hydrate	commercial
Catechol-*O*-sulfate	synthesized
Corilagin	commercial
p-Coumaric acid	commercial
Cyanidin	commercial
Cyanidin-3-*O*-glucoside chloride (Kuromanin chloride)	commercial
Cyanidin-3-*O*-rutinoside chloride	commercial
Cyanidin-3-*O*-sophoroside chloride	commercial
Deoxycholic acid (Cholanoic acid)	commercial
Ferulic acid	commercial
(-)-Epicatechin	commercial
(-)-Epicatechin-3-*O*-sulfate	synthesized
Fisetin (5-Deoxyquercetin)	commercial
Gallic acid	commercial
Guaiacol	commercial
Hydroxytyrosol-3-*O*-sulfate and Hydroxytyrosol-4-*O*-sulfate	synthesized
Hyperoside (Quercetin-3-*O*-galactoside)	commercial
Kaempferol	commercial
Luteolin	MedChemExpress, Monmouth Junction, NJ, USA
3-Methylcatechol	commercial
4-Methylcatechol	commercial

4-Methylcatechol-1-*O*-sulfate and 4-Methylcatechol-2-O-sulfate	synthesized
4-*O*-Methylgallic acid (4'-*O*-Methylgallic acid)	commercial
4-O-Methylgallic acid-3-*O*-sulfate	synthesized
1-*O*-Methylpyrogallol-2-*O*-sulfate	synthesized
2-*O*-Methylpyrogallol-1-*O*-sulfate	synthesized
Myricetin	commercial
Myricetin-3′-*O*-glucoside	commercial
Myricetin-3-*O*-galactoside	commercial
Parthenolide	commercial
Pelargonidin chloride	commercial
Pelargonidin-3,5-di-*O*-glucoside chloride (Pelargonin chloride)	commercial
Pelargonidin-3-*O*-glucoside chloride (Callistephin chloride)	commercial
Phloroglucinol (1,3,5-Trihydroxybenzene)	commercial
o-Phthalaldehyde	commercial
Protocatechuic acid (PCA; 3,4-Dihydroxybenzoic acid)	commercial
Protocatechuic acid-3-*O*-sulfate	synthesized
Pterostilbene (3,5-Dimethyl-resveratrol)	commercial
Pyrocatechol (1,2-Dihydroxybenzene)	commercial
Pyrogallol-1-*O*-sulfate and Pyrogallol 2-*O*-sulfate	synthesized
Quercetin	commercial
Quercetin-*O*-acetyl	synthesized
Salidroside	commercial
Silibinin (Silybin A and Silybin B)	commercial
trans-Resveratrol (3,5,4'-Trihydroxystilbene)	commercial
Tributyrin	commercial
2',4',6'-Trihydroxyacetophenone (2-Acetylphloroglucinol)	commercial
2,3,4-Trihydroxybenzaldehyde	commercial
Tyrosol (2-(4-Hydroxyphenyl)ethanol)	commercial
Vanillic acid-4-*O*-sulfate (3-Methoxybenzoic acid-4-sulfate)	synthesized
**Compounds for structure-activity relationship analysis**	
3′,4′-Dihydroxyflavone	Extrasynthese, Genay, France;
3′,5′-Dihydroxyflavone	Sigma-Aldrich, St. Louis, MO, USA
7,8-Dihydroxyflavone	Sigma-Aldrich, St. Louis, MO, USA
Flavone	Sigma-Aldrich, St. Louis, MO, USA
(+)-Taxifolin (*trans*-Dihydroquercetin)	Sigma-Aldrich, St. Louis, MO, USA
2,3,4,4′-Tetrahydroxychalcone (Butein)	AK Scientific, Union City, CA, USA
3′,4′,7,8-Tetrahydroxyflavone	Extrasynthese, Genay, France
4′,5,6,7-Tetrahydroxyflavone (Scutellarein)	AK Scientific, Union City, CA, USA
3′,4′,7-Trihydroxyflavone	Extrasynthese, Genay, France

Other compounds used	
Enterobactin	Sigma-Aldrich, St. Louis, MO, USA
Ferrosine	Sigma-Aldrich, St. Louis, MO, USA

[1]Compounds were either commercially purchased or synthesized [44]. Suppliers are indicated or to be provided upon request.

Table S3. List of plasmids used.

Plasmid	Source or reference
pFL44-FET4 (from FL44-based genomic bank)	This study
YEplac181	[46]
YEp181-FET4	This study
pRS425-P_{GPD}	[47]
pRS425-P_{GPD}-FET4*	This study
pFET3-lacZ	[48]
pCTH2-lacZ	[48]
pKA475 (*vps13*Δ::*URA3*)	K. Ayscough, University of Sheffield, laboratory collection
